# Whey Protein, Leucine- and Vitamin-D-Enriched Oral Nutritional Supplementation for the Treatment of Sarcopenia

**DOI:** 10.3390/nu14071524

**Published:** 2022-04-06

**Authors:** Emanuele Cereda, Roberto Pisati, Mariangela Rondanelli, Riccardo Caccialanza

**Affiliations:** 1 Clinical Nutrition and Dietetics Unit, Fondazione IRCCS Policlinico San Matteo, 27100 Pavia, Italy; rob.pisati@gmail.com (R.P.); r.caccialanza@smatteo.pv.it (R.C.); 2 IRCCS Mondino Foundation, 27100 Pavia, Italy; mariangela.rondanelli@unipv.it; 3 Unit of Human and Clinical Nutrition, Department of Public Health, Experimental and Forensic Medicine, University of Pavia, 27100 Pavia, Italy

**Keywords:** sarcopenia, muscle mass, muscle protein synthesis, muscle strength, physical performance, nutritional support, whey protein, leucine, vitamin D, oral nutritional supplement (ONS)

## Abstract

Sarcopenia has been recognized as a muscle disease, with adverse consequences on health. Updated recommendations, aimed at increasing awareness of sarcopenia and its accompanying risks, have been produced to urge the early detection and treatment of this disease. Recommended treatment is based on an individually tailored resistance exercise training program, the optimization of protein intake using high-quality protein sources (i.e., whey protein) in order to provide a high amount of essential amino acids—particularly leucine—and addressing vitamin D deficiency/insufficiency. The purpose of this review is to collate and describe all of the relevant efficacy studies carried out with a muscle-targeted oral nutritional supplementation (MT-ONS)—namely a whey-protein-based, leucine- and vitamin D-enriched formula aimed at optimizing their intake and satisfying their requirements—in different patient populations and clinical settings in order to determine if there is enough evidence to recommend prescription for the treatment of sarcopenia or its prevention in high-risk patient populations. Trials using a MT-ONS with or without a concomitant physical exercise program were systematically searched (up to June 2021), and those addressing relevant endpoints (muscle mass, physical performance and function) were critically reviewed. In total, 10 articles providing efficacy data from eight trials were identified and narratively reviewed. As far as older patients with sarcopenia are concerned, MT-ONS has been pertinently tested in six clinical trials (duration 4–52 weeks), mostly using a high-quality randomized controlled trial design and demonstrating efficacy in increasing the muscle mass and strength, as well as the physical performance versus iso-caloric placebo or standard practice. Consistent results have been observed in various clinical settings (community, rehabilitation centers, care homes), with or without adjunctive physical exercise programs. A positive effect on markers of inflammation has also been shown. A muscle-protein-sparing effect, with benefits on physical performance and function, has also been demonstrated in patients at risk of losing skeletal muscle mass (three trials), such as older patients undergoing weight loss or intensive rehabilitation programs associated with neurological disability (Parkinson’s disease). MT-ONS has demonstrated not only a significant efficacy in clinical variables, but also a positive impact on healthcare resource consumption in the rehabilitation setting (length of stay and duration of rehabilitation). In summary, MT-ONS, alone or in association with an appropriate exercise program, is an effective therapy for older patients with sarcopenia and should be offered as a first-line treatment, not only to improve clinical outcomes but also to reduce healthcare resource consumption, particularly in patients admitted to a rehabilitation center.

## 1. Introduction

Sarcopenia, now formally recognized as a muscle disease [1], is a progressive and generalized skeletal muscle disorder, characterized by a decrease in muscle strength and mass. Sarcopenia is highly prevalent in aging community adults (5–10%) [2,3,4], in care homes (15–30% of residents) [2,4], in acute care wards (37% of patients) [2,5] and in up to 76% of patients in the rehabilitation setting [6]. Furthermore, the modern sedentary lifestyle poses additional challenges, as obesity and sarcopenia may co-exist in the form of sarcopenic obesity [7]. Sarcopenia is closely linked to malnutrition, aging, immobility and systemic diseases, with inflammation as a key factor of the pathophysiological process (e.g., malignancy and critical illness) [8,9]. Most importantly, it is a clinical condition associated with adverse outcomes. It predisposes one to physical frailty [10,11], is an established risk factor for falls [12] and is a strong predictor of mortality, disability and institutionalization [13,14]. Moreover, a higher risk of incomplete functional recovery in older hip fracture patients with sarcopenia undergoing in-hospital rehabilitation programs has been observed [15].

Most recently, gait speed and grip strength, two diagnostic measures of physical performance and muscle strength for sarcopenia [8], have been associated with risk in various health outcomes, including cardiovascular disease risk and cancer mortality [16,17].

Despite the high burden of sarcopenia on health, quality of life and healthcare costs [18,19], this muscle disease has been overlooked and under-treated, particularly in older adults, or those patients undergoing muscle recovery or rehabilitation following a catabolic disease state. Accordingly, the European Working Group on Sarcopenia in Older People 2 (EWGSOP2) have recently improved the diagnostic process and provided a clear rationale for the selection of diagnostic measures and cut-off points relevant to clinical practice [6]. The goal is to urge those healthcare professionals who manage patients with or at high risk of sarcopenia to take action with respect to early detection and treatment.

The Belgian Working Group on Nutritional Interventions has recently carried out an umbrella review aimed at providing an overview of nutritional interventions for improving the muscle mass, muscle strength and physical performance in patients aged > 65 years [20]. The authors have included 15 systematic reviews of low to moderate quality—of which, a meta-analysis had been conducted in 6 of them—focusing on a broad range of interventions (proteins, essential amino acids [EAAs], leucine, β-hydroxy-β-methylbutyrate, creatine and multi-nutrient supplementation, with or without physical exercise) investigated in studies that are sometimes of varying quality. Furthermore, the efficacy on sarcopenia has been clearly addressed in a single review among those included. As a conclusion, the most sound evidence available is the recommendation of leucine supplementation in older people with sarcopenia in order to improve muscle mass [20,21]. The low-quality evidence sustaining the use of heterogeneous oral nutritional interventions in combination with exercise is also the main finding of another systematic review addressing the efficacy of treatments in nutritionally vulnerable older adults [22]. A high interstudy variability, with a consequent need to establish the optimal strategy, applies to all major outcomes (muscle mass, muscle strength/function and physical performance) and is likely due to the type, dose, duration and frequency of administration [23]. Therefore, a focused review on a recommended muscle-targeted intervention—namely a whey protein, leucine- and vitamin-D-enriched formula—for sarcopenia or its prevention in high-risk populations was warranted [8,24,25].

## 2. Pathophysiology of the Aging Muscle and Rationale for Nutritional Therapy

Aging is physiologically associated with a reduction in muscle mass: roughly 8% every ten years after the age of 40 and 15% after 70 years [2]. This is likely due to an increased anabolic resistance (a blunted muscle protein synthesis response to anabolic stimuli), a reduced level of physical activity and a decrease in dietary protein intake [8,24,26,27]. Aging is also associated with a reduced availability of amino acids (AAs) due to their increased splanchnic extraction [25]. Furthermore, in older adults, a reduction in muscle protein synthesis (MPS) by 30% has been observed after short-term bed rest or hospitalization, along with a rapid and marked loss of muscle mass (approximately 1 kg of lean body mass in 3 days vs. 0.5 kg in healthy young adults after 28 days) [28].

Several pharmacological approaches are currently under investigation—such as selective androgen receptor modulators and drugs targeting the myostatin-activin pathway—to improve muscle anabolism [27]. However, it is clear that optimized nutrition should be considered as a standard of care, as the availability of the building blocks needed for muscle mass recovery is critical. Accordingly, in order to stop and possibly reverse the loss of muscle mass and function, the current guideline-based recommended treatment consists of resistance exercise training, protein intake optimization and addressing vitamin D deficiency/insufficiency [8,24,25]. Nutritional recommendations for an older adult (>65 years) population propose an increase in daily protein intake (1–1.2 g/kg/day; 1.2–1.5 g/kg/day in case of inflammatory disease), preferably of high-quality protein (i.e., whey protein), containing large amounts of essential amino acids (EAAs) such as leucine [24,25]. More specifically, to overcome the anabolic resistance of the aging muscle and maximize MPS throughout the day, 25–30 g of high-quality protein and up to 2.8–3 g of leucine should be given at each meal and at least twice daily (minimum suggested intake of leucine, 78.5 mg/kg/day) [24,29,30]. Accordingly, in order to reach nutrient anabolic thresholds, high-quality oral nutritional supplementation (ONS) should be considered in the presence of inadequate food intake. Whey protein has proven to be a valuable protein source resulting in greater anabolic stimulation due to faster digestion and a higher content of EAAs compared to other protein sources [31]. Among the EAAs, leucine has proved to be a potent and independent modulator of protein turnover, particularly of protein anabolism [29,32].

Finally, also taking the endemic deficiency in older adults into account, concomitant vitamin D supplementation should be considered (at least 800–1000 IU/day). Vitamin D has multiple genomic and non-genomic effects on the muscle (i.e., regulation of cell cycle gene expression, differentiation of muscle cells and protection against senescence replication) [33]. Furthermore, it has been demonstrated to be synergic with leucine in potentiating protein anabolism [34], with a potential benefit on muscle function, particularly in advanced age, in the presence of insufficient serum levels and in combination with physical activity [35,36,37].

With this background, a critical review of studies investigating the benefits of a muscle-targeted nutritional approach—namely a whey protein-based, leucine- and vitamin-D-enriched formula—is timely. Despite being a recommended treatment strategy and a reasonable standard of care for all patients with sarcopenia, the impact of muscle-targeted ONS (MT-ONS) has never been the focus of critical appraisal. This is relevant given the heterogeneous efficacy of standard nutritional approaches—with or without physical exercise—in broader patient populations in whom the presence of sarcopenia has not been specifically addressed [20,22,23]. This applies to muscle mass, muscle strength/function and physical performance, with a high interstudy variability due to the type, dose, duration and frequency of administration, which suggests that the optimal strategy is yet to be established [23].

In the present review, available data on the use of MT-ONS were summarized to potentially characterize an evidence generation process. In order to justify the focus on MT-ONS, we first addressed nutrikinetic and nutridynamic studies in healthy and sarcopenic patients dealing with the absorption of key nutrients—namely whey protein and leucine (regardless of a combination with vitamin D)—in plasma in the post-administration state, as well as their distribution and metabolism within the body, which underpin a thorough investigation of efficacy. Attention was then focused on efficacy data from clinical trials—either randomized or not—undertaken in patient populations diagnosed with sarcopenia—based on a validated diagnostic process—or at a high risk of developing it (e.g., patients undergoing weight loss programs or intensive physical rehabilitation programs for neurological disability). To address this last issue, English-language intervention studies using a whey protein-based formula (>80% of total protein content from whey), enriched with leucine and vitamin D with or without a concomitant physical exercise program were systematically searched (up to 30 June 2021) through electronic databases (PubMed, Embase, and Scopus). Therefore, only trials addressing efficacy endpoints relevant to the topic (muscle mass, physical performance and function) were thoroughly reviewed and critically evaluated according to the setting of care and the research hypothesis (treatment or prevention of sarcopenia).

## 3. Nutrikinetic and Nutridynamic Studies

**Figure 1:**
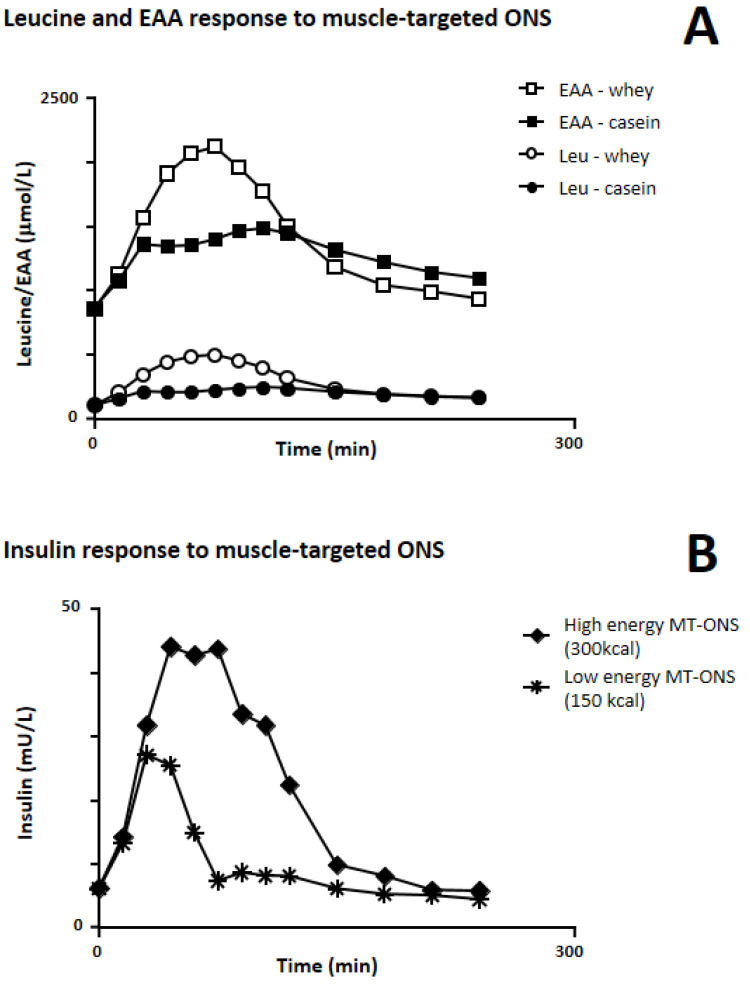
Serum levels (in healthy older subjects) of: (**A**) leucine (Leu) and essential amino acids (EAA) in response to the ingestion of a low-calorie (150 kcal) casein-based and leucine-enriched whey-protein-based (muscle-targeted) ONS; (**B**) insulin in response to the ingestion of a low-calorie (150 kcal) and a high-calorie (300 kcal) muscle-targeted ONS (MT-ONS). Adapted from the study by Luiking et al. [39].

Several robust nutrikinetic and nutridynamic studies (Table 1) have demonstrated that oral supplementation with key nutrients—namely whey-protein-based ONS either enriched or not enriched with leucine, which is primarily involved in muscle anabolism—produces the highest post-prandial plasma concentrations of AAs (Figure 1A) and stimulates MPS rates in both sarcopenic and non-sarcopenic subjects to a greater extent than any other protein source independently of the concomitant provision of energy and the combination with resistance exercise training.

In one study, initially addressing only the quality of the protein source, postprandial absorption kinetics and the muscle protein fractional synthesis rate (FSR) were examined in 48 healthy older men (mean age 74 years) who were randomly assigned to ingest 20 g of labelled whey protein (WP), casein or casein hydrolysate. The peak appearance rate of AAs in the circulation was higher with WP than with casein or casein hydrolysate. Similarly, FSR values were higher after WP than after C and CH ingestion [38].

A proof-of-principle study was also performed to evaluate the acute effect of a standardized breakfast supplemented with a MT-ONS on the postprandial MPS of healthy older men (*n* = 24; Table 2). Subjects (mean age 71 years) were randomly allocated, in a double-blind fashion, to the test supplement or to a non-caloric placebo. At first administration (week 0; 0–240 min), the postprandial FSR was higher in the test group than in controls [43]. The acute effect of a single bolus of a high WP, leucine-enriched supplement on MPS compared with an isocaloric milk protein control and its combined effect with resistance exercise was also evaluated in a randomized, controlled, double-blind trial in 19 healthy older adults. This trial was completed using a unilateral resistance exercise protocol. Results showed that the postprandial muscle protein FSR, immediately after exercise training, was significantly higher after the intervention product vs. the control, most likely due to higher postprandial concentrations of EAAs and leucine [39].

To study the impact of macronutrient composition on postprandial serum AA profiles, 12 healthy older subjects were randomized (single-blind, cross-over design) to receive four iso-nitrogenous (21 g) supplements with different amounts of energy (leucine-enriched WP with 150/320 kcal; and C with 150/320 kcal). Peak concentrations of leucine, EAAs and total AAs were two-fold higher for WP150 vs. C150, higher for WP320 vs. C320 and higher for low-energy vs. high-energy products, and the co-administration of energy (Figure 1B) resulted in a greater postprandial rise in insulin concentration [40]. In another study [41] conducted to address the effect of the macronutrient composition on MPS, 45 non-sarcopenic older men (mean age 69 years) were randomly assigned to receive a protein-energy supplementation (21g of leucine-enriched WP with 9 g of carbohydrates and 3 g of fats) or protein (21 g of leucine-enriched WP) or energy (an iso-caloric mixture of carbohydrates and fats) alone. The ingestion of protein with or without energy significantly increased the postprandial muscle protein FSR vs. basal, whereas energy alone had no effect. Despite a greater postprandial rise in circulating insulin concentration occurring with the co-administration of energy, no difference in MPS was observed between the two groups receiving WP, demonstrating that the addition of carbohydrates and fats did not significantly alter the MPS response, at least in healthy subjects [41].

To evaluate whether the MPS rate differs between sarcopenic and non-sarcopenic older men, 15 healthy men (mean age 69 years) and 15 sarcopenic men (mean age 81 years) received a single bolus of a leucine-enriched WP nutritional supplement (21 g protein). Basal and postprandial muscle protein FSR were measured using a stable isotope methodology and the collection of blood and muscle samples. Following protein ingestion, the FSR increased significantly in both groups, with no between-group differences [42].

Indeed, considering that the implementation of a normal food-based diet represents the first-line strategy, there is an additional question that should be addressed. Are EAAs plasma levels with a WP nutritional supplement different from what can be obtained following protein ingestion through normal food? A randomized study conducted in 66 older adult malnourished individuals admitted to a rehabilitation unit and comparing the provision of dietary proteins between a “spread” diet (SD, i.e., dietary protein intake spread over four daily meals) and a “pulse” diet (PD, i.e., 72% of dietary protein—averaging 1.31 g/kg body weight daily—given in one meal at noon) demonstrated that PD, despite being more efficient than SD [51], yields approximately a 50% lower increase in the plasma postprandial concentration of EAAs than a single bolus of a WP, leucine-enriched supplement (20 g of WP and 2.8 g of leucine) [38,39,40,41]. Therefore, it is reasonable to argue that daily and single-meal recommended protein targets could be achieved only through the consumption of a high-quality protein source, and more efficiently obtained with the combined use of a high-quality ONS.

These studies may be considered proof-of-concept trials, already showing the effect of a muscle-targeted oral nutritional supplement (ONS) on MPS. Such stimulation of muscle protein synthesis paves the way for a wider clinical development program, aimed at demonstrating the efficacy of MT-ONS not only on muscle mass, but also on physical performance and function. Nonetheless, although vitamin D has pleiotropic effects and the optimization of intake is recommended in patients with sarcopenia [8,24,25,33], its role in muscle anabolism and function/performance still needs to be clarified. Indeed, multiple trials have addressed the impact of its supplementation on different muscle outcomes (mass, strength and power), but only a small benefit on muscle strength has been demonstrated, with a higher and clinically meaningful efficacy in specific subgroups of patients, such as those aged ≥ 65 years and those presenting serum insufficiency (<30 nmol/L) [35]. On the other hand, while there is more convincing evidence that vitamin D has anabolic properties in myotubes and rodents, including a synergic stimulation with leucine [34,52], findings on an independent effect on protein synthesis in humans are inconsistent. In an 8-week double-blind placebo-controlled RCT (interventions: (1) vitamin D, 2000 IU/day; (2) conjugated linoleic acid, 4000 mg/day; (3) both nutrients; (4) placebo [corn oil]) conducted in 32 sedentary older adults (age range, 60–85 years) with suboptimal serum vitamin D (<35 ng/mL), Van Vliet and colleagues reported no effect of supplementation on MPS or handgrip strength [44]. Therefore, it is reasonable to argue that the value of vitamin D in improving muscle outcomes is closely related to and dependent on appropriate nutritional repletion and nutrient intake optimization, such as an adequate intake of proteins and EEAs.

## 4. Efficacy Trials

Successful proof-of-concept studies have been followed by clinically meaningful RCTs [45] (Table 2 and Table 3).

In total, the search identified 47 non-duplicated, potentially eligible articles. After excluding 21 papers on the grounds of a review of their titles and abstracts, 26 full-text articles were examined, and 10 articles providing efficacy data from eight trials were identified and narratively reviewed (Supplementary Figure S1). These studies, although of heterogeneous duration (range 4–52 weeks), have addressed and demonstrated the efficacy of MT-ONS not only on muscle mass—recovery or sparing—but also on measures of performance strength and physical function, as these outcome measures are far more relevant for this patient population [8,27]. The efficacy has been tested with or without a standardized exercise program, depending on the setting of care. Other outcome data (e.g., healthcare resource consumption, inflammation, protein and energy intake) have also been collected and interpreted as being relevant to the support of a further improvement of patient care.

### 4.1. MT-ONS in the Community Setting

A multi-center, randomized, placebo-controlled, double-blind, parallel group trial (the PROVIDE study) examined the effect of MT-ONS on measures of sarcopenia.

Three hundred and eighty non-malnourished older patients with sarcopenia (mean age 78 years, 65% female, 88% living independently) with Short Physical Performance Battery (SPPB; 0–12) scores of 4–9 associated with a low skeletal muscle mass index (SMMI) were randomized to MT-ONS (*n* = 184) per serving or to an isocaloric control product containing carbohydrate and fat (*n* = 196), given twice daily for 13 weeks. Although the trial did not reach a significant between-group difference in co-primary efficacy variables (SPPB and grip strength), the chair stand test, a component of SPPB and a measure of lower extremity function, as well as appendicular muscle mass, showed a significant improvement in patients on muscle-targeted ONS vs. control group [46].

The PROVIDE study generated secondary analyses as well. The first one, conducted to evaluate whether baseline serum 25-hydroxy vitamin D [25(OH)D] and the amount of dietary protein intake influence changes in muscle mass and function, demonstrated that sufficient baseline levels of 25(OH)D (at least 50 nmol/L) and dietary protein intake (at least 1 g/kg/day) are needed to respond more efficiently to a nutritional strategy aimed at attenuating muscle loss in sarcopenic older patients [55]. Vitamin D and WP have demonstrated an impact on inflammation markers [57,58]. Furthermore, taking into account the relevance of inflammation (chronic low-grade or acute disease-related) in the pathophysiology of sarcopenia [11], in a second post hoc analysis, the levels of several circulating markers (IL-8, IL-1 receptor antagonist, soluble TNF receptor, IL-6 and high-sensitivity C-reactive protein) were assessed, showing that the use of a MT-ONS led, after 13 weeks, to an attenuated progression of chronic low-grade inflammation [48]. A third analysis has also detected a small but significant benefit of MT-ONS on markers of bone health (parathyroid hormone [↓], carboxy-terminal collagen crosslinks [↓] and bone mass density [↑]) [50].

Finally, in the study performed by Chanet et al. to evaluate the effect of a standardized breakfast supplemented with a MT-ONS in healthy older men (*n* = 24), a significant benefit toward appendicular lean mass measured by dual-energy X-ray absorptiometry (predominantly as leg lean mass) was observed at the end of the intervention period (week 6) [43].

### 4.2. MT-ONS in Rehabilitation Units and Care Homes

This clinical setting allows for the evaluation of the combination of MT-ONS with a supervised physical exercise program in patients taking standard institutional meals.

In a double-blind, controlled, parallel-group trial, 130 sarcopenic older patients (mean age 80 years) admitted to a rehabilitation clinic were randomized to consume a MT-ONS (1 serving/day) or an isocaloric amount of maltodextrin for 12 weeks. Nutritional interventions were provided in association with a comprehensive individualized training program of moderate intensity for 12 weeks designed to improve physical fitness and muscle mass. At the end of the follow-up period, a significant increase in lean body mass (fat-free mass and relative skeletal muscle mass [SMM], measured by dual-energy X-ray absorptiometry) and handgrip strength, as well as physical function (standardized summary scores for physical components and activities of daily living), were observed in the MT-ONS vs. control group [54]. Furthermore, CRP levels were lowered and QoL scores were improved by the muscle-targeted formula.

The efficacy of MT-ONS (two servings/day) on the outcome of a physical exercise rehab program in 140 older in-patients with sarcopenia was also compared with an isocaloric control formula in a 4–8 week randomized, double-blind, controlled study (the IRIS study). The primary efficacy endpoint—the difference in 4 m gait speed per month—was significantly better in the MT-ONS group. Likewise, key secondary endpoints related to physical performance measures reached statistically and clinically significant improvements: the chair stand test, TUG test and SPPB. All other efficacy outcome variables (Barthel index, handgrip strength, ADL, QoL and appendicular muscle mass) with the exception of the quality of life (SF-12) were also significantly improved. CRP levels were lowered only with the use of the muscle-targeted formula, which was also associated with reduced healthcare resource consumption, as derived by a shorter duration of the rehabilitation program and length of stay (approximately 10 days) [49].

These results were consistent with those of another trial conducted in parkinsonian patients—majority at a high risk of developing sarcopenia (*n* = 150; prevalence of sarcopenia 2%)—who underwent a 30-day multidisciplinary intensive rehabilitation treatment (PRO-LEADER study). This was a pragmatic, randomized, assessor blind, controlled trial comparing a MT-ONS (two servings scheduled during the day to avoid interference of levodopa absorption with protein ingestion) vs. standard of care. The primary endpoint—6 min walking distance—as well as relevant secondary endpoints addressing muscle performance (i.e., 4 m walking speed and timed up and go [TUG]) were improved after 30 days of MT-ONS administration. Furthermore, a significant sparing effect on SMM was observed in patients receiving the MT-ONS, whereas non-supplemented patients experienced a decrease in muscle mass [47]. No adjustment of concomitant levodopa dosing schedule was necessary, which was a clinically important advantage for PD patients.

Finally, a single-arm trial was performed in a care home, where all residents (*n* = 95) were screened for the presence of sarcopenia (prevalence 85%). Among these, 39 had an evaluable functional status and were prescribed a MT-ONS (two servings/day) according to a challenge–dechallenge–rechallenge study design. Twenty-two out of thirty-nine residents were eligible for a supervised physical exercise rehabilitation program for 12 months, whereas 17 patients took only the MT-ONS. After 6 months, the WP exercise cohort showed an increase in SMM and handgrip strength, as well as an improvement in gait speed and SPPB score, whereas the WP-only cohort exhibited an increase in SMM, but not in handgrip strength. These advantages were no longer present after 3 months of therapy and were restored after 3 months back on therapy [56], thus suggesting the importance of the continuity of muscle-targeted nutritional support.

### 4.3. MT-ONS in Sarcopenic Obesity

Most recently, greater interest has risen in sarcopenia associated with obesity [7]. Weight loss may be beneficial even in advanced age but it may be accompanied by the loss of skeletal muscle mass, which may accelerate the development of sarcopenia. Therefore, therapy should focus on minimizing the loss of muscle mass. One high-quality study addressing muscle preservation has been conducted in 80 obese old adults undergoing a 13-week weight loss program (hypocaloric diet with an energy deficit of 600 kcal/day) in conjunction with resistance training (three times/week). Patients (mean age 63 years; mean BMI 33 kg/m^2^) were randomized, according to a double-blind parallel group design, to receive a MT-ONS or an isocaloric control (10 servings/week; 7 just before breakfast and 3 after the exercise sessions). At the end of the 13-week observation period, both groups achieved a significant reduction in body weight and fat mass, with no between-group differences. However, an increase in appendicular muscle mass was detected in the MT-ONS group, whereas a decrease in the same variable was observed in the control group. The muscle strength and physical performance, as assessed by the handgrip strength and 4 m gait speed, 400 m walk speed and chair stand, respectively, improved in both groups [53].

These results were confirmed by another lifestyle intervention study (PROBE study) conducted by the same research group and using the same design and intervention in subjects with type 2 diabetes (*n* = 123). Although the body weight and fat mass were reduced in both groups (no between-group difference), in the whole study population (*n* = 123), the use of a MT-ONS improved the total and appendicular skeletal muscle mass, with a trend to significance for leg mass, whereas no effect was detected on the physical performance and function outcome measures. A significant effect on fasting insulin and insulin sensitivity and resistance was also detected [59]. The effect of supplementation with MT-ONS was also examined in the subgroup of study-compliant subjects (*n* = 82) according to the presence of muscle insulin resistance. Only in patients with insulin resistance (*n* = 42) did the use of a MT-ONS (*n* = 20) improve the appendicular skeletal muscle mass, whereas no effect was detected on the knee extension power and leg press strength [60].

## 5. Discussion and Conclusion

**Figure 2:**
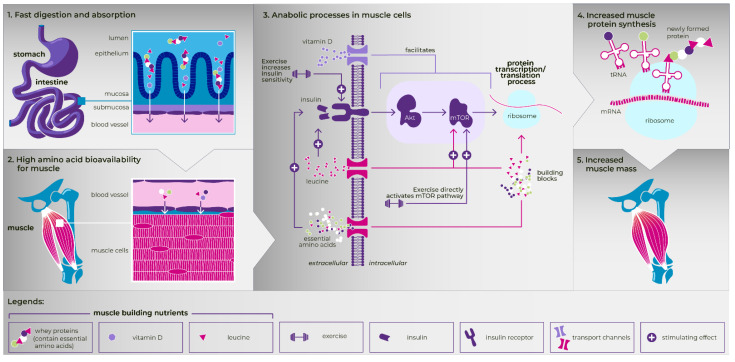
The effect of muscle-targeted ONS (whey protein, leucine and vitamin D) in combination with exercise in increasing appendicular muscle mass in older adults with sarcopenia (the present figure is used with permission from Danone Nutricia Research BV for this single publication).

Practicing physicians need reliable evidence from RCTs regarding which treatments will benefit their patients the most. Several studies have been conducted with MT-ONS, and most of them are of high quality. Results consistently support the use of MT-ONS, preferably in combination with an exercise program, as an ideal intervention to promote MPS, increase muscle mass and strength, and improve the physical performance and physical function of older patients with sarcopenia, as well as to preserve the muscle mass in patients at high risk of developing it (Figure 2).

Evidence of efficacy in our review has been obtained by: (1) studying a clearly defined patient population, which is not always the case in clinical nutrition trials; (2) using a homogeneous MT-ONS—classified as a food for special medical purposes—given at a standard dosage (two servings/day) in most trials; (3) and addressing outcome measures related to muscle mass and physical performance/function, which are clinically meaningful and relevant for appropriately addressing this disease condition [8,27].

The efficacy of MT-ONS was found to be higher in association with physical exercise [47,49,53,54,56], but nevertheless also evident in non-exercising patients [45,48,56]. This is a clear advantage for this patient population, in which, access to physical rehabilitation may be limited (e.g., due to clinical reasons, resource availability, logistics, etc.). Nonetheless, in parkinsonian patients—most of whom could be non-sarcopenic but at risk of sarcopenia—the muscle overuse linked to rigidity, and, in advanced stages of disease, also to involuntary movements, may lead to muscle loss, which may be further exacerbated by intensive rehabilitation programs. In fact, these patients randomized to standard dietary care during intensive rehabilitation in fact lost muscle mass, despite improving their physical performance, whereas patients treated with MT-ONS maintained it, as well as had an improved performance compared to the control group [54]. The same muscle-protein-sparing benefit, reasonably associated with improved insulin sensitivity (and anabolic resistance), has been confirmed in obese older adults requiring lifestyle modification to reduce body weight [53,59,60].

The robustness of the data is further highlighted by its generalizability, as the efficacy of MT-ONS has been demonstrated in different settings and real-life heterogeneous patient populations, with a high burden of co-morbidities due to the avoidance of stringent inclusion criteria [61,62]. Furthermore, MT-ONS has been shown to reduce healthcare resource consumption in rehabilitation, translating clinically relevant improvements into valuable savings for the healthcare system [49].

The main strength of this review is its focus on the selection of studies testing a well-defined MT-ONS as a treatment of patients with or at high risk of sarcopenia, avoiding the heterogeneity of nutritional formulas and patient populations, making the data-driven recommendations strong and reliable. Although the limited number of trials retrieved could be a limitation, the relatively homogeneous results have led to clear-cut conclusions. Another limitation is the inclusion of English-language trials only, although a recent meta-epidemiologic study found that excluding non-English publications from reviews on clinical interventions had a minimal effect on overall conclusions [63]. We were also not able to define an optimal duration of the intervention. To detect an effect, including the recovery of muscle mass, a minimum duration should be 4-8 weeks, although a continuous maintenance dose could be reasonably hypothesized. Indeed, future research should address the efficacy and the tolerability of long-term supplementation, namely beyond 6 months given on a daily basis (two servings/day), or as a cyclic administration. Data on its tolerability (gastro-intestinal tolerability, kidney function and vitamin D and calcium toxicity) up to 6 months have been provided [56,64,65]. Furthermore, the trial conducted by Dimori et al. [56] has shown that a 3-month interruption after 6 months of continuous administration of MT-ONS resulted in a loss of efficacy, which then recovered after a further 3 months of MT-ONS intake. The study was observational (challenge–dechallenge–rechallenge study design) and the topic warrants a RCT in order to draw firm conclusions.

Some additional unresolved issues remain. The synergistic effect of the MT-ONS, given in association with a physical exercise program outside a rehabilitation setting, deserves an in-depth evaluation. The same applies to the potential existence of gender-related differences in efficacy. Most nutrikinetic and nutridynamic studies have included male participants and this issue has never been addressed in efficacy trials. A more specific focus on patient populations characterized by substantial muscle wasting should be considered. Trials in this area are lacking and the identification of populations gaining the most benefits from the intervention could have important implications at both the clinical and health economic level. Analyses of cost-effectiveness could be relevant as well.

Finally, the optimization of energy intake should also be taken into account and addressed in the near future. This could ensure the best possible clinical and functional recovery in this frail patient population. It has been suggested that trials addressing the efficacy of drugs for the treatment of sarcopenia should not include patients with severe malnutrition [27]. Nonetheless, malnutrition and sarcopenia are substantially overlapping syndromes [9]. With this perspective, the IRIS trial has shown that most patients admitted to a rehabilitation setting suffer from malnutrition (mean Mini Nutritional Assessment score of approximately 18 points), and it has suggested that, despite the satisfactory optimization of protein intake in patients receiving MT-ONS (mean intake 1.1 g/kg/day) and an increase in energy intake in both study arms, older patients frequently did not reach the minimum suggested energy target of 27–30 kcal/kg/day [49]. Therefore, in future studies, the use of a high-energy MT-ONS in a malnourished population, potentially in combination with physical exercise, is reasonable and warranted. Based on trials reviewed herein, the use of a MT-ONS with a relatively low energy content has consistent evidence of efficacy in addressing the recovery of muscle mass and function. On the other hand, the consumption of a high-energy formula has been found to enhance insulin secretion, resulting in an improved muscle protein turnover (increased synthesis and reduced breakdown) [40,66]. Higher energy provision could prevent dietary proteins being oxidized as an energy source, but adding energy to the formula could hamper the appetite and the intake of the nutritional supplement, whilst the additional energy may lower the amino acid peak of the protein that is consumed [40]. Nonetheless, additional energy is not needed, and may even be detrimental for overweight and obese (not energy-malnourished) people [53,59,60].

In conclusion: there is sufficient evidence to recommend a muscle-targeted oral nutritional supplementation as a first-line nutritional treatment of sarcopenia, most likely combined with a tailored physical exercise program to further enhance clinical outcomes. Its use in the prevention of sarcopenia in high-risk populations should be considered as well.

## Figures and Tables

**Table 1:** Main characteristics of nutrikinetic and nutridynamic studies.

Author, Year [Ref]	Study Aim	Study Design	Participants	Experimental Intervention (Dosages)	Control Intervention (None or Description)	Combined Physical Activity Intervention (None or Description)	Findings	Other Findings
Pennings, 2011 [38]	To compare protein digestion and absorption kinetics and post-prandial muscle protein accretion after ingestion of different protein sources	Randomized, parallel-group trial	Healthy older men (*n* = 48; age, 74 ± 1 years)	Single bolus of whey protein (20 g)	Single bolus of casein (20 g) or casein hydrolisate (20 g)	None (avoidance any sort of exhaustive physical activity for 3 days before the experiment)	− Peak appearance rate of dietary protein-derived labeled panylalanine in the circulation: greater with whey protein and casein hydrolisate than with casein (*p* < 0.05) − Fractional synthesis rate (FSR): higher after whey protein than casein and casein hydrolisate (*p* < 0.05)	Strong positive correlation (r = 0.66; *p* < 0.01) between peak plasma leucine concentration and post-prandial FSR
Luiking, 2014 [39]	To evaluate muscle protein synthesis after ingestion of two different oral nutritional supplements (ONS) and to study the combined effect with resistance exercise, using a unilateral resistance exercise protocol.	Randomized, parallel-group, double-blind trial	Healthy older adults (*n* = 19; males, 47%; age, 69 ± 6 years)	Single bolus of whey protein (20 g) leucine-enriched (3 g) supplement	Conventional iso-caloric diary product (single bolus containing 6 g of proteins)	Unilateral resistance exercise protocol	FSR: higher after whey protein + leucine vs. control (*p* = 0.049)	None
Luiking, 2016 [40]	To evaluate the impact of ONS with distinct protein source and energy density on serum amino acids (AAs) profile	Randomized, cross-over, single-blind trial	Healthy adults (*n* = 12; males, 42%; age, 67 ± 2 years)	Single bolus of low-calorie (150 kcal) and high-calorie (300 kcal) whey-protein-based (20 g) ONS	Single bolus of low-calorie (150 kcal) and high-calorie (300 kcal) casein-based (20 g) ONS	None	− Peak serum leucine concentrations: 2-fold higher for low-calorie whey protein ONS vs. low-calorie casein ONS (*p* < 0.001); higher for high-calorie whey protein ONS vs. high-calorie casein ONS (*p* < 0.001); higher for pooled low-calorie ONS vs. pooled high-calorie ONS (*p* < 0.001) − Peak concentration of essential AAs and total AAs: comparable to that of leucine	In vitro digestion modelling for 90 min resulted in higher levels of free total AAs, essential AAs and leucine for low-calorie whey protein ONS vs. low-calorie casein ONS, for low-calorie whey protein ONS vs. high-calorie whey protein ONS, and for low-calorie casein ONS vs. high-calorie casein ONS. High-calorie ONS resulted in higher serum insulin concentration vs. low-calorie ONS (*p* < 0.001)
Kramer, 2015 [41]	To determine the impact of the macronutrient composition of ONS on the post-prandial muscle protein synthesis (MPS) rates	Randomized, parallel-group, double-blind trial	Non sarcopenic older men (*n* = 45; age, 69 ± 1 years)	Single bolus of two different isonitrogenous whey protein (20 g) leucine-enriched ONS containing (150 kcal) or not containing carbohydrate and fat	Protein-free isocaloric mixture (150 kcal) containing carbohydrate and fat	None	− MPS: significantly increased only after ONS containing protein-leucine (vs. baseline, *p* < 0.05); between-group comparison significant only for protein-leucine ONS containing calories vs. protein-free ONS (*p* = 0.01)	Insulin levels: greater post-prandial rise after protein-leucine ONS containing calories, but not significantly superior to ONS containing protein-leucine only
Kramer, 2017 [42]	To assess basal and post-prandial muscle protein FSR in healthy and sarcopenic subjects	Comparative study of two different patients populations	Healthy adults (*n* = 15; age, 69 ± 1 years) and sarcopenic older men (*n* = 15; age, 81 ± 1 years)	Single bolus of a low-calorie (150 kcal) whey protein (20 g) leucine-enriched ONS	None	None	− Muscle protein FSR: significantly increased in both sarcopenic (*p* = 0.003) and healthy subjects (*p* < 0.001) compared to baseline, with no between-group difference during the early and late stages of the post-prandial period	None

**Table 2:** Risk of bias of the clinical trials included in the review.

Author, Year	Random Sequence Generation (Selection Bias)	Allocation Concealment (Selection Bias)	Blinding of Participants and Personnel (Performance Bias)	Blinding of Outcome Assessment (Detection Bias)	Incomplete Outcome Data (Attrition Bias)	Selective Reporting (Reporting Bias)	Other Bias
*PROVIDE study* Bauer, 2015 [44] Verlaan, 2018 [45] Liberman, 2019 [46]	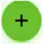	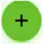	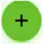	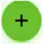	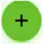	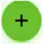	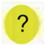
Verreijen, 2015 [47]	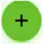	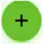	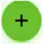	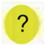	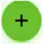	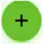	Single-center
Rondanelli, 2016 [48]	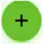	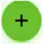	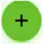	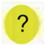	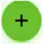	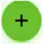	Single-center
Chanet, 2017 [43]	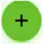	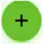	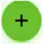	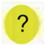	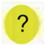	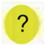	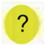
Dimori, 2018 [49]	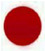	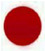	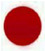	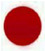	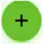	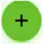	Single-center
*PRO-LEADER study* Barichella, 2019 [48]	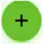	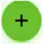	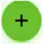	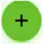	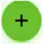	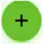	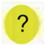
*IRIS study* Rondanelli, 2020 [50]	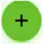	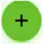	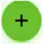	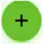	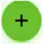	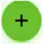	Single-center

+ is “good quality”; red stands for “high risk of bias”; yellow stands for “information not reported”.

**Table 3:** Main characteristics of trials addressing the efficacy of muscle-targeted oral nutritional supplementation.

Author, Year [Ref]	Study Design	Setting	Study Duration	Muscle-Targeted Intervention (Dosages)	Control Intervention (None or Description)	Combined Physical Activity Intervention (None or Description)	Muscle Mass	Physical Performance Endpoints	Physical Function Endpoints	Other Endpoints
Bauer, 2015 [46] PROVIDE study (first analysis)	RCT, multi-centre	Community	13 weeks	Twice daily (21 g whey protein, 3 g leucine and 800 IU vitamin D each serving) for 13 weeks	Isocaloric matched placebo	None	Appendicular muscle mass (Between-group difference of 0.17 kg; *p* = 0.045)	***Handgrip strength*** (No between- group differences)	***SPPB*** (No between- group differences) Chair stand test (Delta = −1.01 s, *p* = 0.018); gait speed; balance score	None
Verreijen, 2015 [53]	RCT, single-centre	Community	13 weeks	10 times/week (21 g whey protein, 3 g leucine and 800 IU of vitamin D each serving) for 13 weeks	Isocaloric matched placebo	Resistance training 3X/week for 13 weeks in both groups	***Appendicular muscle mass*** (+0.4 kg vs. −0.5 kg; *p* = 0.03)	Handgrip strength (No between-group differences)	400 m walking test; 4 m gait speed test; chair stand test	Body composition
Rondanelli 2016 [54]	RCT, single-centre	Rehabilitation center	12 weeks	Once daily (22 g whey protein, 4 g leucine and 100 IU of vitamin D each serving)	Isocaloric matched placebo	Controlled physical activity program (20 min exercise session/day, 5 times/week)	***Fat free mass*** (1.7 kg gain; *p <*0.001); relative skeletal muscle mass (*p* = 0.009)	Handgrip strength (improved with test product; *p* = 0.001)	Activities of daily living	Body composition; IGF-1 and PCR; HR-QoL; global nutritional status
Chanet, 2017 [43]	RCT, single-centre	Community	6 weeks	Once daily before breakfast (21 g whey protein, 3 g leucine and 800 IU of vitamin D each serving) for 6 weeks	Non caloric flavored watery placebo	None	***Mixed muscle protein synthesis rate****(FSR)* (higher in the test group; *p* = 0.001); appendicular lean mass (higher in the test group; *p* = 0.035)	Handgrip strength (No between- group differences)	SPPB (no between-group differences)	Body composition; blood glucose, insulin, EAA and leucine
Verlaan, 2018 [55] PROVIDE study (secondary analysis)	RCT, multi-centre; post hoc analysis	Community	13 weeks	Twice daily (21 g whey protein, 3 g leucine and 800 IU vitamin D each serving) for 13 weeks	Isocaloric matched placebo	None	Appendicular muscle mass (higher baseline concentrations of 25(OH)D are associated with greater gain in AMM)	None	Chair stand test (no effect of baseline concentrations of 25(OH)D)	None
Dimori, 2018 [56]	Observational study: cross-sectional survey (Phase 1) + single-arm intervention trial (Phase 2)	Care home	6 months on + 3 months off + 3 months on	Twice daily (21 g whey protein, 3 g leucine and 800 IU vitamin D each serving) when administered	None	Patients with Tinetti score >9: 40 min physical therapy session, 3 times/week for 12 months	Skeletal muscle mass	Handgrip strength	SPPB (patients with Tinetti score > 9); gait speed (4 m walking test)	Body composition; sarcopenia prevalence (Phase 1 of the study)
Liberman, 2019 [48] PROVIDE study (Tertiary analysis)	RCT, multi-centre	Community	13 weeks	Twice daily (21 g whey protein, 3 g leucine and 800 IU vitamin D each serving) for 13 weeks	Isocaloric matched placebo	None				IL-8 (higher decrease with the test product; *p* = 0.03); IL-1RA and IL-6 (no significant between-group differences); sTNFR1; CRP; pre-albumin
Barichella, 2019 [47] PRO-LEADER study	RCT, pragmatic, bicentric, assessor-blind	Rehabilitation centre for patients with Parkinson’s disease	30 days	Twice daily (21 g whey protein, 3 g leucine and 800 IU vitamin D each serving) for 30 days	Usual care	Multidisciplinary Intensive Rehabilitation Program (MIRT)	Skeletal muscle mass (increased vs. usual care; *p* = 0.029) and skeletal muscle index	Handgrip strength	***6 min walking test*** (+18.1 m vs. usual care; *p* = 0.039); 4 m walking speed, timed up and go, Berg balance scale (all improved vs. usual care)	
Rondanelli, 2020 [49] IRIS study	RCT, single-centre	Rehabilitation centre	Until discharge (at least 4 weeks and up to 8 weeks)	Twice daily (21 g whey protein, 3 g LEU and 800 IU vit. D each serving) for 4-8 weeks	Isocaloric control formula	Controlled physical activity program (20 min exercise session/day, 5 times/week)	Muscle mass (increased vs. control; *p <*0.03)	Handgrip strength (increased vs. control; *p <*0.03)	***Change in4 m gait speed/month*** (+0.063 m/sec/month with active vs. control; *p <*0.001); chair stand test; timed up and go test; SPPB (all improved vs. control; *p <*0.001)	Cognitive function tests (both improved vs. control; *p <*0.001); rehabilitation intensity profile (improved vs. control; *p* = 0.003); probability of being discharged at home (higher vs. control; *p* = 0.002); overall economic benefit (duration of rehabilitation and length of hospital stay, both improved vs. control; *p <*0.001)

Abbreviations: RCT, randomized clinical trial. The primary endpoint is highlighted in bold italic. *p*-values for effect were reported where available.

## Supplementary Materials

The following supporting information can be downloaded at: https://www.mdpi.com/article/10.3390/nu14071524/s1, Figure S1: PRISMA flow-chart.
