# Supplementary figures and images for: Whey Protein, Leucine- and Vitamin-D-Enriched Oral Nutritional Supplementation for the Treatment of Sarcopenia
Source: Nutrients. 2022 Apr 6;14(7):1524. doi: 10.3390/nu14071524 (PMC9003251; doi:10.3390/nu14071524)

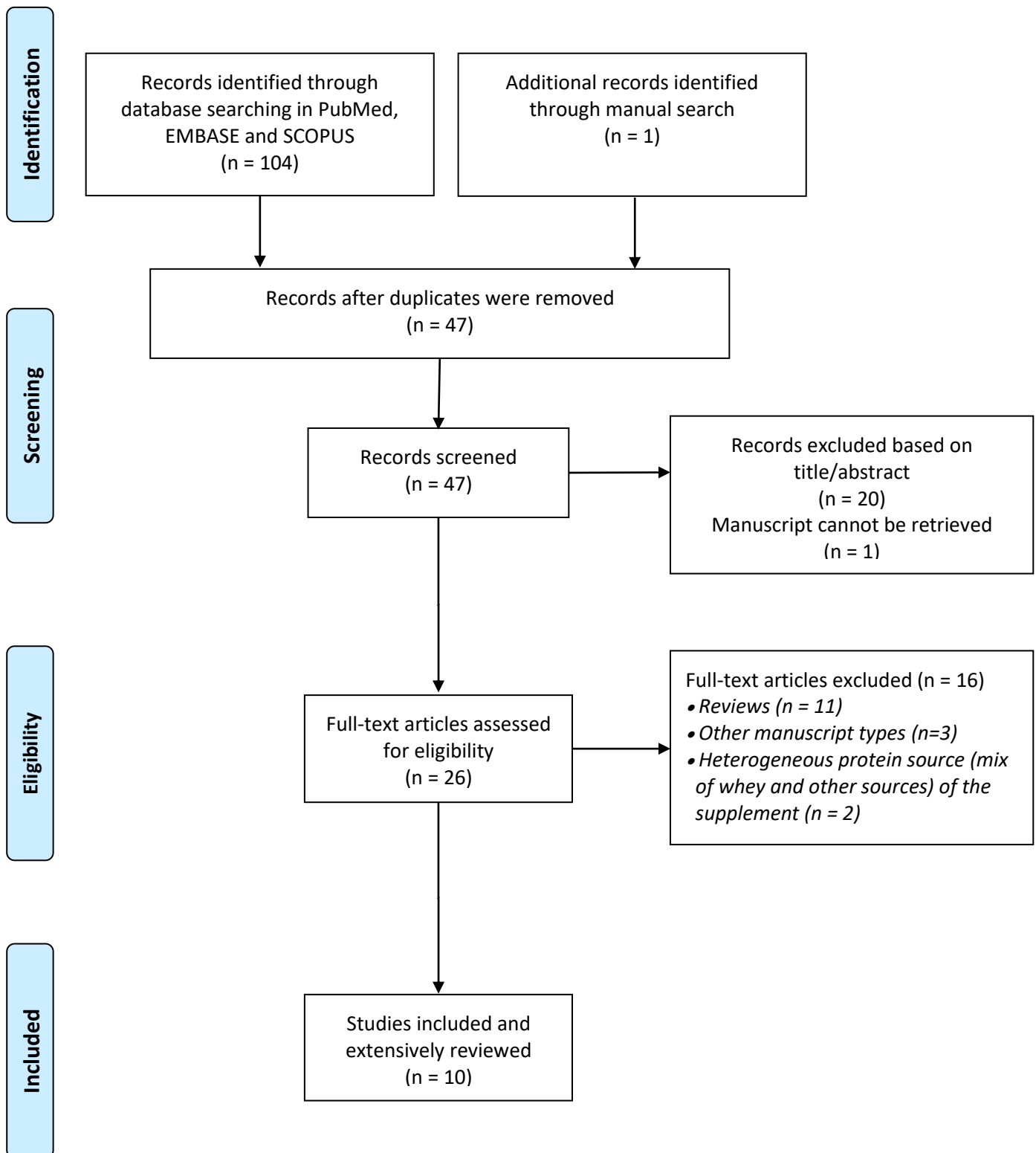

**Supplementary Figure S1.** PRISMA flow-chart.

Supplement: Supplementary file 1 [file nutrients-14-01524-s001.zip › nutrients-1637789-Supplementary.pdf]
